# A new perspective on immune evasion: escaping immune surveillance by inactivating tumor suppressors

**DOI:** 10.1038/s41392-022-00875-6

**Published:** 2022-01-13

**Authors:** Svenja Mergener, Samuel Peña-Llopis

**Affiliations:** 1 Translational Genomics in Solid Tumors, German Cancer Consortium (DKTK) and German Cancer Research Center (DKFZ) at University Hospital Essen, Hufelandstrasse 55, D-45147 Essen, Germany; 2 Division of Solid Tumor Translational Oncology, German Cancer Consortium (DKTK) and German Cancer Research Center (DKFZ), D-69120 Heidelberg, Germany

**Keywords:** Cancer genetics, Tumour immunology

Inactivating mutations in tumor suppressor genes (TSGs) are known to drive tumorigenesis by decoupling proliferation and survival from tight regulatory mechanisms. In a recent study published in *Science*, Martin et al.^1^ shed light on an unappreciated role of TSGs in preventing immune evasion by demonstrating enrichment of TSG inactivation in tumor cell transplants in mice when facing an intact adaptive immune system.

The capacity of the immune system to recognize and eliminate initiatory cancer cells has been subject to critical discussions throughout the twentieth century. In recent decades, various preclinical and epidemiological studies validated the immune system’s function as a barrier to the formation of neoplasms.^2^ As a consequence, transformed cells managing to form tumors in immunocompetent organisms must be able to escape immune surveillance, constituting an essential step in the complex process of tumorigenesis. Hence, understanding these immune evasion mechanisms has the potential to build a path to novel therapeutic strategies by disrupting such adaptations, leading to the recognition and elimination of cancer cells by inherent immune cells. Present attempts to restore eradication of tumor cells by the immune system mainly focus on the principle of immune checkpoint blockade, typically by blocking cytotoxic T lymphocyte-associated protein 4 (CTLA-4) or programmed cell death protein 1 (PD-1). The expression of ligands to immune checkpoint receptors like CTLA-4 (CD80, CD86) or PD-1 (PD-L1, PD-L2) on the tumor cell surface mediates inhibition of T-cell responses, representing one way of immune evasion that can be pharmaceutically targeted.^3^ Despite the successful use of present immune checkpoint inhibitors in several cancer entities, many cancer types do not respond to such compounds, suggesting distinct mechanisms of immune evasion that still need to be elucidated.

**Fig. 1:**
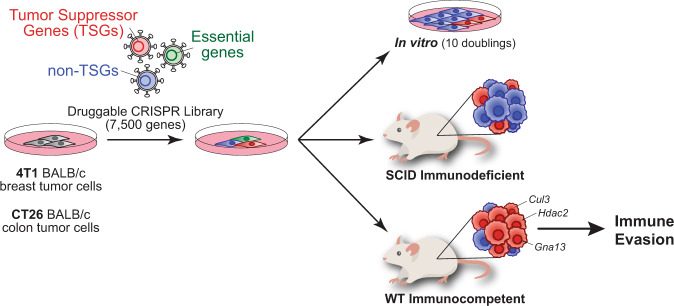
The inactivation of tumor suppressor genes (TSGs) fosters immune evasion to promote tumorigenesis. BALB/c tumor cells from the breast (4T1) and colon (CT26) were screened with a druggable CRISPR library targeting ~7500 genes. As expected, cells with loss of essential genes (depicted in green) were dropped out from the screen after ten population doublings in vitro or in vivo. However, cells knocking out TSGs (depicted in red) were largely enriched in tumors from cells implanted subcutaneously on flanks of wild-type (WT) immunocompetent mice compared to SCID immunodeficient mice or cells grown in vitro. These results highlight a new role of the loss of TSGs in evading immune surveillance to support tumor survival and growth.

In this study, Martin et al.^1^ performed CRISPR screens using a library of single-guide RNAs (sgRNAs) targeting around 7500 druggable genes, which they used for single copy transduction in mouse tumor cell lines of breast and colon cancer. The researchers transplanted the transduced cell lines in two mouse strains with different immunological states—wild-type BALB/c mice with an intact adaptive immune system and immunocompromised SCID BALB/c mice—and compared sgRNA abundance in the resulting tumors in order to identify genes facilitating tumor growth in their respective immune microenvironments. Here, they found a remarkable overrepresentation of sgRNAs targeting TSGs previously known to play important roles in human carcinogenesis in immunocompetent relative to immunocompromised mice (Fig. 1). These findings suggest the requirement of the presence of an adaptive immune system and elucidate a new aspect of TSGs by demonstrating that TSG loss can result in immune evasion.

Previous studies mainly focused on TSG functions related to controlled cell death, DNA damage repair, protein degradation, cell differentiation, migration, and angiogenesis, among others,^4^ thereby leading to proliferation and survival advantages related to their inherent impact on tumor cells themselves upon TSG loss. However, little is known about how TSG loss impacts the interaction between initiating tumor cells and the host immune system. The work by Martin et al.^1^ shows a low overlap between the TSGs identified in breast and colon cancer cell lines (13%), as well as a low overlap with the most common TSGs mutated in some human cancers, indicating that the role of TSGs in evading immune surveillance is tissue context-dependent. Moreover, by utilizing a sgRNA library targeting a broad spectrum of genes and selective analysis of tumor cells capable of escaping the adaptive immune system, Martin et al. demonstrate the value of CRISPR screening approaches to identify such previously unappreciated genes in terms of their involvement in immune surveillance mechanisms. In another study, Burr et al.^5^ were able to identify a previously unknown major regulator of PD-L1 expression in cancer cells, *CMTM6*, by making use of a whole-genome CRISPR library screen. In order to identify regulators of PD-L1 in tumor cells, Burr and colleagues transduced a PD-L1-expressing pancreatic cancer cell line with a CRISPR library targeting all protein-coding human genes, followed by expansion and selective analysis of rare cells with low PD-L1 expression. Subsequent analyses in PD-L1-expressing melanoma, breast- and lung cancer cell lines confirmed *CMTM6* to be an important regulator of PD-L1, providing further evidence on how CRISPR library screens can facilitate the uncovering of immune escape mechanisms. Similarly, Martin et al.^1^ unraveled the regulatory role of one of the enriched TSGs in their study, namely *guanine nucleotide binding protein a3* (*Gna13*), in cancer immune escape mechanisms by modulating the tumor microenvironment. By further investigation of Gna13 in colon cancer cells, the authors found *Gna13* to negatively regulate the expression of the chemokine *Ccl2/MCP-1*, resulting in an increased secretion of CCL2 upon loss of GNA13, which led to the recruitment of pro-tumor macrophages (TAMs) to the tumor microenvironment. Interestingly, converse depletion of CCL2 led to a decrease in tumor size and TAM recruitment only in the background of GNA13 loss, but not in GNA13 wild-type tumors, proposing pharmaceutical targeting of CCL2 in *GNA13*-mutant cancers as a possible therapeutic intervention to be further investigated. These studies demonstrate the potential of identifying key regulators in cancer immune escape mechanisms to open the avenue for developing novel therapies, which could complement standard immune checkpoint inhibitors targeting CTLA-4 or PD-1 or other therapeutic agents.

Taken together, the work of Martin et al.^1^ offers important insights into how TSG loss can affect immune evasion, which poses an emerging field of research with the potential of developing novel therapeutic approaches in cancer immunotherapy. By connecting the advantages of CRISPR-based library screens and a fully functional adaptive immune system in vivo, the authors provide new perspectives in cancer immune research and emphasize the limitations of conventional in vitro studies regarding the investigation of oncogenic drivers in the context of the immune system as well as the importance of models that include the tumor immune microenvironment, allowing a holistic comprehension beyond sole phenotypic changes in tumor cells. Further studies using similar approaches will shed light on detailed mechanisms that drive cancer immune evasion and will hopefully help to understand and overcome cancer progression by restoring immunologic processes.
